# Addressing Data Absenteeism and Technology Chauvinism in the Use of Gamified Wearable Gloves Among Older Adults: Moderated Usability Study

**DOI:** 10.2196/47600

**Published:** 2024-04-24

**Authors:** Edmund W J Lee, Warrick W Tan, Ben Tan Phat Pham, Ariffin Kawaja, Yin-Leng Theng

**Affiliations:** 1 Wee Kim Wee School of Communication and Information Nanyang Technological University, Singapore Singapore Singapore; 2 Centre for Information Integrity and the Internet Nanyang Technological University, Singapore Singapore Singapore; 3 Ageing Research Institute for Society and Education Nanyang Technological University, Singapore Singapore Singapore; 4 StretchSkin Technologies Pte Ltd Singapore Singapore; 5 SingHealth Polyclinics Singapore Singapore

**Keywords:** wearables, exergames, older adults, active aging, rehabilitation, stroke

## Abstract

**Background:**

Digital health technologies have the potential to improve health outcomes for older adults, especially for those recovering from stroke. However, there are challenges to developing these technologies, such as *data absenteeism* (where older adults’ views are often underrepresented in research and development) and *technology chauvinism* (the belief that sophisticated technology alone is the panacea to addressing health problems), which hinder their effectiveness.

**Objective:**

In this study, we aimed to address these challenges by developing a wearable glove integrated with culturally relevant exergames to motivate older adults to exercise and, for those recovering from stroke, to adhere to rehabilitation.

**Methods:**

We conducted a moderated usability study with 19 older adults, of which 11 (58%) had a history of stroke. Our participants engaged in a 30-minute gameplay session with the wearable glove integrated with exergames, followed by a quantitative survey and an in-depth interview. We used descriptive analysis to compare responses to the System Usability Scale between those who had a history of stroke and those who did not. In addition, we analyzed the qualitative interviews using a bottom-up thematic analysis to identify key themes related to the motivations and barriers regarding the use of wearable gloves for rehabilitation and exercise.

**Results:**

Our study generated several key insights. First, making the exergames exciting and challenging could improve exercise and rehabilitation motivation, but it could also have a boomerang effect, where participants may become demotivated if the games were very challenging. Second, the comfort and ease of use of the wearable gloves were important for older adults, regardless of their stroke history. Third, for older adults with a history of stroke, the functionality and purpose of the wearable glove were important in helping them with specific exercise movements.

**Conclusions:**

Our findings highlight the importance of providing contextual support for the effective use of digital technologies, particularly for older adults recovering from stroke. In addition to technology and usability factors, other contextual factors such as gamification and social support (from occupational therapists or caregivers) should be considered to provide a comprehensive approach to addressing health problems. To overcome data absenteeism and technology chauvinism, it is important to develop digital health technologies that are tailored to the needs of underserved communities. Our study provides valuable insights for the development of digital health technologies that can motivate older adults recovering from stroke to exercise and adhere to rehabilitation.

## Introduction

### Background

There has been an increasing trend in the development and implementation of digital health technologies for older adults, known as *gerontechnologies*. However, there are challenges to developing these technologies, such as *data absenteeism* (where older adults’ views are often underrepresented in research and development) and *technology chauvinism* (the belief that sophisticated technology alone is the panacea to addressing health problems) [1]. Gerontechnologies aim to assist older adults in healthy aging through the promotion of physical exercise and empowering them to maintain a certain level of functional independence throughout their later years and delay the onset of frailty [2,3]. For instance, examples of such technologies are the use of *exergames* to motivate physical activity or virtual reality technology for the purposes of pain management and therapy [4,5]. Other types of health technologies, such as wearables, are also becoming common among older adults. Existing commercial products such as FitBit (Google, Inc) and Apple Watch make it easy for older adults to track and monitor their health [6]. To date, such digital health technologies targeted at older adults have received substantial attention and investments, ranging from US $1.1 billion to US $3.1 billion between 2019 and 2020, and it is expected to grow in the next few years [7,8].

While there are several studies documenting the positive impact of digital health technologies on older adults’ physical and mental health, there are 2 key gaps in existing public health literature [9,10]. First, very few studies have explicitly addressed the problems of *data absenteeism* and *technology chauvinism.* Second, as many of the existing health technologies are developed in a clinical setting for data collection, few researchers have examined how to develop technology that is fun, relatable, and equitable for the older adults [11]. The overall objective of the study was to address the problem of data absenteeism and technology chauvinism in the development of digital health technologies—a stretchable wearable glove integrated with exergames—by examining the motivations and barriers of older adults without a history of stroke and those recovering from stroke in the use of such technologies through the lens of equity.

### Theoretical Framework: Data Absenteeism and Technology Chauvinism

While the momentum to build and develop different digital health technologies by academia or industry is noteworthy, it is important to adopt the lens of equity in building such technologies for older adults. Older adults are often disadvantaged as compared to the general and younger population in terms of their access, use, attention, and processing of health information from digital health technologies [12]. Not giving attention to the context in which such digital health technologies are developed and introduced may result in the unintentional consequence of exacerbating the health disparities between those who are well resourced and those with less resources [13]. In advocating for technology and big data use through an equitable lens, Lee and Viswanath [1] argued for the need for health communication and informatics scholars and technology developers to pay attention to the 2 perennial problems: data absenteeism and technology chauvinism.

*Data absenteeism* refers to a situation where data from underserved populations are not represented. For example, recent studies of large-scale national programs that use wearables to boost physical activity, such as Singapore’s National Steps Challenge, which offers free wearables and gamified mobile apps to encourage participants to walk 10,000 steps, show that older adults are often underrepresented in the data. This gap aligns with previous findings indicating that users of such technology tend to be younger and more educated and possess a higher degree of eHealth literacy [14,15].

In contrast, *technology chauvinism*, refers to the blind faith in big data systems or technology platforms in addressing health disparities. One of the most infamous cases was the use of Google Flu Trends to predict the outbreak of influenza, where search trends overestimated the prevalence of influenza as compared to official sources [16,17]. To address the problems of data absenteeism and technology chauvinism in the use of digital health technologies to improve physical activity among healthy older adults and those recovering from stroke, it is vital to involve them from the start of the research process and engage them to codevelop wearable gloves and exergames. This is consistent with studies of the principles of user-centered design, where it is crucial to be intentionally inclusive in the process by incorporating the target group as full partners in the decision and design groups as part of the research process. This ensures that their participation and input are not merely symbolic or exploitative, but rather meaningful and beneficial for them as a whole [18].

### Context of the Study: Motivating Rehabilitation and Exercise Using Wearable Gloves and Exergames

In recent years, there has been a surge in the exploration and creation of wearable glove technologies aimed at enhancing exercise motivation and aiding stroke recovery. The smart glove industry is projected to reach a value of US $3.9 billion by 2028, with an annual growth rate of 10% [19]. These innovative gloves are developed in 2 main styles: rigid hand exoskeletons and soft assistive gloves. The latest soft rehabilitation gloves are being designed to support bending, straightening, and spreading or closing of each finger to address the difficulties some patients face in performing hand grabbing motion [20].

There are several existing studies that have documented the efficacy of the use of wearable gloves for patients with stroke in improving upper limb movement across several metrics. For instance, Yurkewich et al [21] tested the Hand Extension Robot Orthosis (HERO) Grip Glove among 11 participants with difficulty in finger extension in their poststroke journey and found that the glove significantly improved their water bottle grasp and index finger movement and extension and enabled individuals who are lacking grip strength to handle blocks, use a fork, and write with a pen. Wang et al [22] conducted a study where 69 patients with severe upper limb impairment following a stroke were divided into three treatment groups: (1) repetitive transcranial magnetic stimulation, (2) soft robotic glove use, and (3) standard treatment, and the results showed that the group assigned to use the robotic gloves achieved better upper extremity scores compared to those in the standard treatment group.

Given that the use of wearable gloves is comparable to neurostimulation or neuromodulation techniques in improving movements for patients with stroke, other research teams have incorporated the use of other forms of stimulation (ie, tactile sensations) in wearable gloves to improve its efficacy. Seim et al [23] conducted a pilot study involving 16 patients with chronic stroke, where participants were randomly placed into 2 groups for an 8-week period: one group received a vibrotactile stimulation glove, and the other group received a similar glove without vibration (acting as the control condition). The outcomes demonstrated that those using the vibrotactile stimulation glove experienced notable enhancements in finger mobility and improvements in the range of motion of their elbows and shoulders compared to the control group.

While these studies have found improvements in upper limb movements through the use of wearable gloves in their respective sample, it was unclear why or how they were effective. In a systematic review of wearable technology for improving activity in adult patients with stroke, Parker et al [24] found that, overall, very few studies have found evidence for the use of wearable gloves to improve rehabilitation. Thus, a significant oversight in many recent advancements in these state-of-the-art wearable gloves is the lack of consideration for how their target user group might engage with these technologies and the probability of their adoption as consumer products. Examples of these gaps in knowledge would be ideas about what could motivate a potential consumer to purchase the wearable glove product and incorporate it into their daily rehabilitation routine, and ultimately, what could be the barriers that prevent them from making the gloves a staple part of their rehabilitation journey. Examining how specific design features affect the perceived usefulness and perceived ease of use of the product could also affect their decision to adopt these fancy wearable gloves into their routines [25].

### Addressing the Gaps in Wearable Gloves Research: Integrating Wearable Gloves With Exergames

One of the ways to improve rehabilitation through wearable gloves among older adults is through the gamification of the rehabilitation process. This is done through the use of specialized video games that simulate exercise, known colloquially as *exergames*. It is an area of health technology that has gained interest in recent years, especially among older adults. According to Harrington et al [26], the use of exergames may address barriers to older adults being physically active and exercising. These video games demand that the player physically moves their body to advance within the game or program [27]. Therefore, the implications surrounding the movement-centric nature of the gaming technology means that it has the potential to be used in rehabilitative health care practices also.

Existing systems such as the Nintendo Wii Fit, a low-cost commercial gaming system, have been found to be effective in improving clinical measures of balance in older adult patients [28,29]. Thus, exergames are able to transform exercise, through the process of gamification, by introducing alternative motivators such as entertainment and encouragement into activities that would otherwise be considered as physically strenuous [30]. A study by Yu et al [31] found that exergames as an intervention using the Xbox Kinect significantly improved the physical activity level, leg strength, and cardiopulmonary endurance of healthy older adults. Although exergames make physical exercise and rehabilitation more accessible, there are still obstacles to the uptake of these innovations in health technology among older adults.

One key aspect in which exergames have been shown to aid the rehabilitation process is adherence to exercise. Research by Oesch et al [32] on the difference between conventional self-regulated exercise and exergames revealed that exergames showed heightened levels of adherence to rehabilitation exercise routines within the first 2 weeks of introduction. However, the same study also showed that the motivation levels reversed after the first 2 weeks.

### Understanding the Motivations and Barriers

One of the main goals of using a gamified wearable glove is to further motivate patients and older adults who are undergoing hand rehabilitation by increasing the movement of their hands through gameplay using wearable gloves and, subsequently, reduce the barriers to use of technology through seamless incorporation of the gaming medium. In addition, the hand movement data that the gloves are capable of collecting can further assist health care professionals in providing focused rehabilitation activities to their individual patients.

As such products are aimed to be used by patients and older adults, one must also consider motivations and barriers to adoption. This is because new technologies (ie, the wearable gloves) may present potential problems from the perspective of the consumers (older adults and health care professionals), such as being incompatible with existing products or technologies and individuals’ needs [33]. In this regard, health care technology has never really seen the introduction of gloves with the technological capability of collecting vast amounts of data from each exergaming session. Traditionally known as “haptic gloves,” these wearable devices offer force feedback and originated in the virtual reality gaming industry. They have the potential for gathering data for video game technology developers, although their application in rehabilitation and health care is quite rare [34]. Thus, to understand older adults’ needs, it is paramount to investigate their motivations and barriers regarding the use of wearable gloves and exergames and whether their past experiences with rehabilitation have any effect on their perception of these products.

### Study Objectives

This study aims to answer the following research questions (RQs) regarding the general usability of these wearable gloves in the context of the rehabilitation process:

- RQ1: What are the motivations and barriers toward the use of an integrated wearable glove system with exergames?
- RQ2: What are the differences between older adults without a history of stroke and older adults with a history of stroke regarding their perceptions about an integrated wearable glove system with exergames?

## Methods

### Wearable Glove and Exergame Development

**Figure 1:**
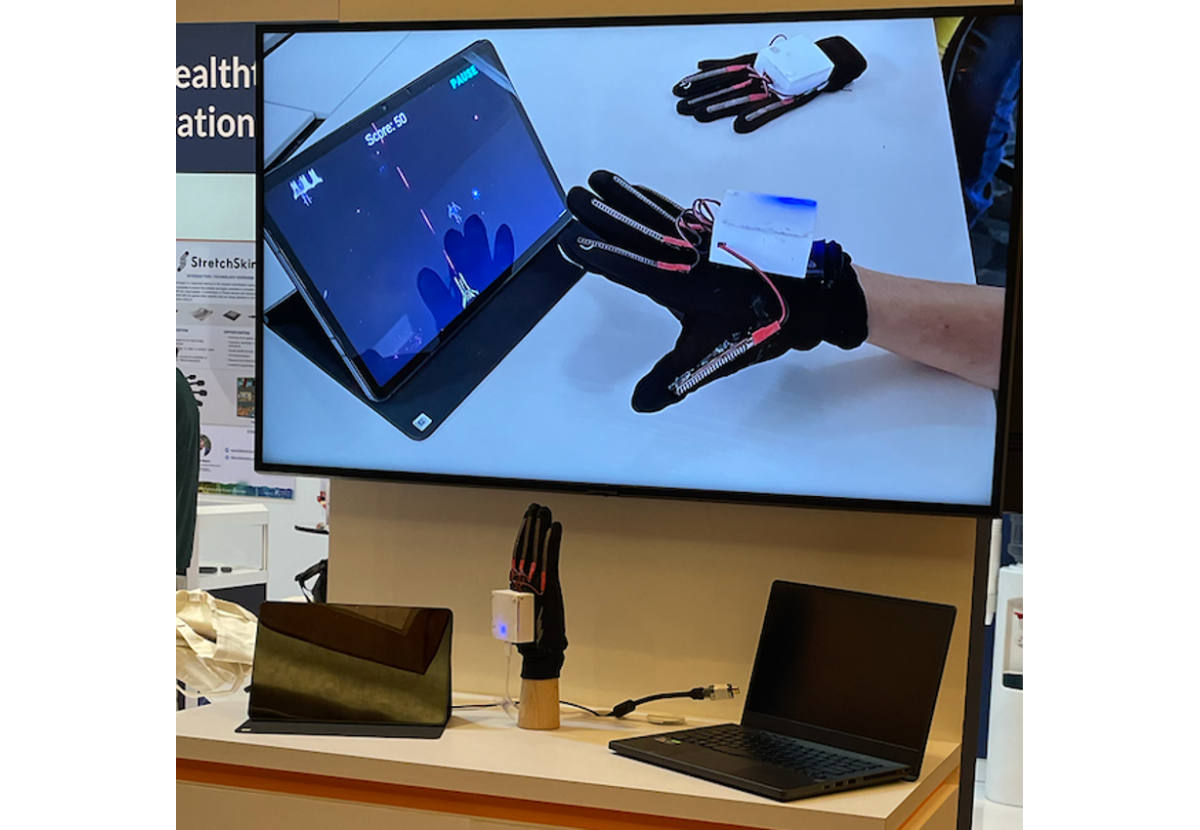
Prototype of the wearable glove.

**Figure 2:**
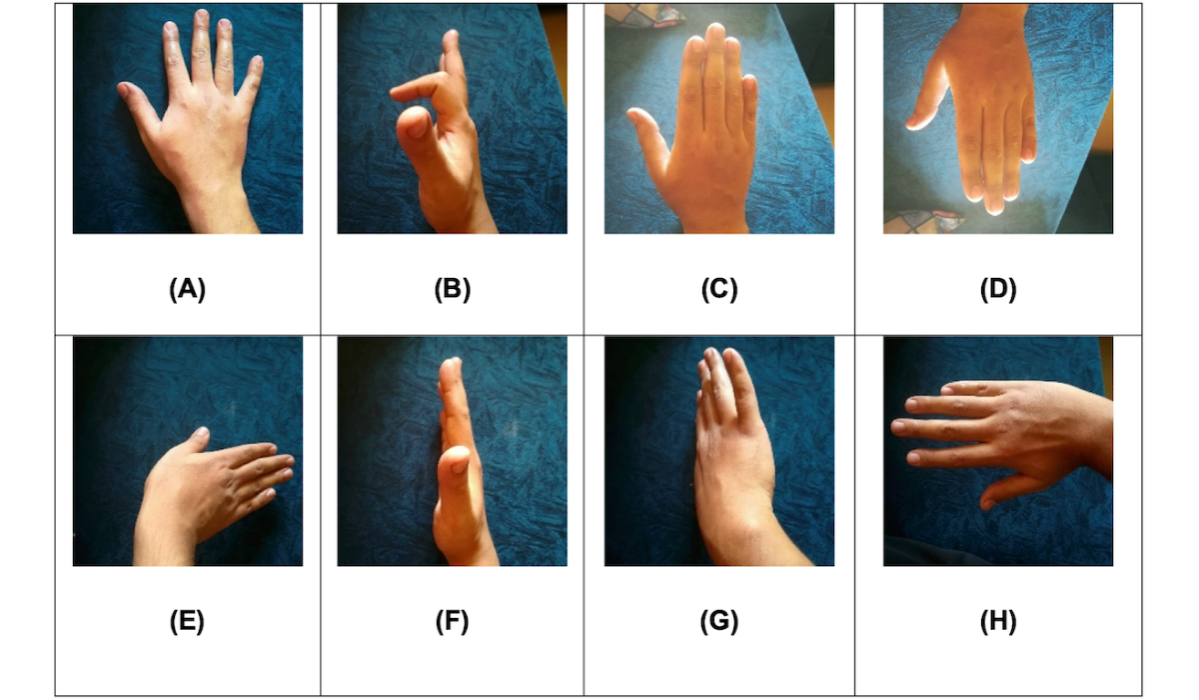
Specific hand motions for rehabilitation: (A) idle, (B) finger trigger, (C) up, (D) down, (E) right side, (F) right, (G) left, and (H) left side.

Before the study, the research team worked with a start-up company that specializes in the development of rehabilitation gloves to develop a wearable data glove that could be integrated with exergames. Figure 1 shows the glove prototype that was developed for both survivors of stroke and older adults, specifically to capture hand motion for gesture recognition and for data visualization with the help of the multiple sensors integrated within the gloves. The bendable and flexible sensors embedded in the glove cover the joints on each individual finger and capture bending data signals from finger movement. This technology supports the training of specific wrist, hand, or finger movement and finger joints mobilization activities that is commonly found in the rehabilitation process, as seen in Figure 2.

The gloves were designed to be integrated with existing in-house exergames developed by the research team at the university. The exergame system consists of exercises and games that could be personalized for each individual user to suit different durations and number of repetitions for a particular exercise [35,36]. These exergames were specifically created to suit older adults to promote successful aging by motivating exercise, where they aim to improve physical and cognitive functional capacity through easy-to-follow actions and interface and culturally relevant game themes [37].

**Figure 3:**
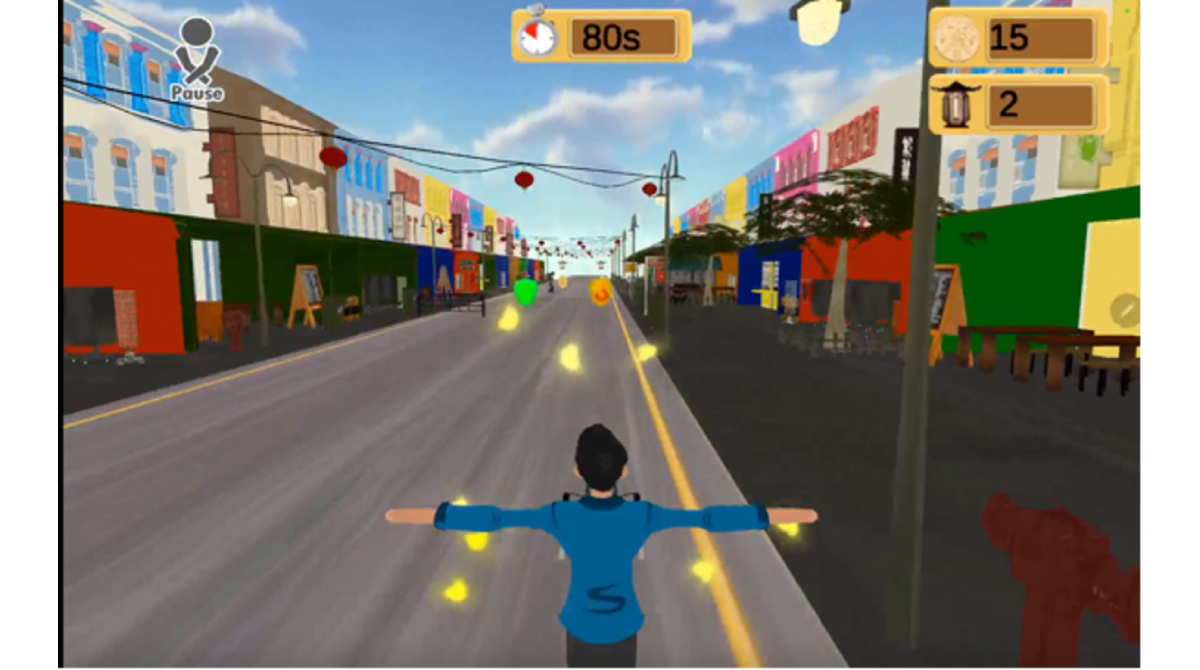
Screenshot of the Chinatown Race gameplay.

While there were a series of 9 exergames developed to date, the research team integrated the wearable glove with 1 exergame called “Chinatown Race” (Figure 3), where players are required to dodge barriers while aiming to collect coins to score points while running down a road in an online Chinatown setting. The avatar is controlled through rotation of hand and finger movements that would be detected by the wearable glove. As shown in Figure 2, participants had to perform movement 5 and movement 8 to slide the avatar toward left and right, respectively. To catch the lantern power-ups in the game, participants had to flex their pointer finger, as seen in movement 2.

### Participants and Recruitment

After the development of the wearable glove and integration with the exergame, we conducted a moderated usability study with 19 participants aged ≥50 years, recruited from an older adult activity center with a convenient sample of individuals with and without a history of stroke (n=11, 58% had a history of stroke and n=8, 42% did not). The average age of the participants was 66.8 (SD 4.5) years, and 53% (10/19) were women and 47% (9/19) were men. Of the 19 participants, 13 (68%) belonged to the Chinese ethnic group, while 4 (21%) belonged to the Malay ethnic group. There was also 5% (1/19) Burmese and 5% (1/19) Singh individuals who participated in the study. Overall, 74% (14/19) of the participants were considered to have lower levels of education below an “A-Level” certification. The inclusion criteria for the study were that participants had to be (1) aged at least 50 years and (2) willing and able to use the wearable glove to play the designated exergames and participate in a qualitative interview and survey. The moderated usability sessions were conducted in either English or Chinese depending on the language preference of the participants.

### Ethical Considerations

Before the study, we obtained approval from the institutional review board of from the institutional review board of Nanyang Technological University, Singapore (IRB-2022-405). Written informed consent was also obtained from the participants. They were briefed about the procedures involved in the use of the wearable glove and exergames; they were also informed that the risks were minimal in the gameplay and that they could exit the study without any penalty. Upon successful completion of all the tasks in the moderated usability study, the participants were given a voucher worth SGD 50 (US $37) as incentive. To safeguard participants' privacy, the data were de-identified before analysis.

### Study Design and Procedure

The moderated usability study session was designed to be a 1-hour long session, which consisted of a series of tasks involving participants’ use of the wearable glove and exergame, led by 1 moderator and 1 technical specialist. The moderator guided the participants through the required activities and conducted the interview and survey upon the completion of the tasks. The technical specialist ensured that the devices were working as intended and handled all the technical difficulties that arose during the session.

In the session, participants were required to complete the following tasks:

1. Navigation of wearable glove and exergames: Participants were taught the basics about how to use the wearable gloves and control functions on the exergame dashboard.
2. Chinatown Race gameplay: Participants had to use the wearable glove to engage in Chinatown Race gameplay, where they had to move their avatars to avoid barriers and collect lanterns to score points by using hand and finger rotation.
3. Qualitative interview: This is a qualitative interview through which the moderator obtained feedback about participants’ attitude and perceptions regarding the wearable glove and exergame.
4. System Usability Scale (SUS) survey: The moderator administered a short SUS scale, which is a 10-item measure of the usability of systems.

### Measures of SUS

The SUS scale (Cronbach α=.95) was adapted from Chu et al [37], and participants provided their responses to the following items measured using a 5-point scale (1=strongly disagree and 5=strongly agree): (1) I think that I would like to use the glove frequently, (2) I found the glove unnecessarily complex, (3) I thought the glove was easy to use, (4) I think that I would need the support of a technical person to be able to use the glove, (5) I found the various functions of the glove to be well integrated with the game, (6) I thought there was too much inconsistency with the glove, (7) I would imagine that most people would learn to use the glove very quickly, (8) I found the glove very cumbersome to use, (9) I felt very confident using the glove, and (10) I needed to learn a lot of things before I could get going with the glove.

The qualitative interviews were analyzed using a bottom-up thematic analysis to identify key themes regarding the motivations and barriers toward the use of wearable gloves for rehabilitation and exercise among older adults who had a history of stroke and those who did not. The thematic analysis was performed in accordance with the steps described by Proulx et al [38] regarding usability testing of wearable gloves: (1) the recordings were transcribed, (2) the transcript was first read by a member of the research team to develop the coding frame, and (3) the coding frame was refined to identify different types of motivators and barriers regarding the use of wearable gloves and exergames. Next, descriptive analysis was conducted to compare the differences in responses to SUS items between older adults who had a history of stroke and those who did not. This is consistent with the studies by Tong et al [39] and Casterlé et al [40].

## Results

### Competition as a Motivation

RQ1 involves the motivations and barriers toward the use of a wearable glove integrated with exergames. Multimedia Appendix 1 shows the summary of the mean scores of participants’ responses to SUS items. From the qualitative interview, one of the key motivations identified was that a certain *degree of competition* was required to ensure that the older adults are engaged and motivated. For instance, the inclusion of features such as collectible coins, lanterns, and a scoring system presented a form of incentive for the participants to continue persevering to complete the game. These features made the game fun, as there was a tangible goal associated with each movement that they made within the game. Some even noted that it spurred them to strive to get better at the game, as a participant mentioned the following:

To score the highest score, that is very exciting.

Participant 10; with a history of stroke

As such, the competitive element of the gameplay objectives plays a key role in motivating older adults to continue playing and, in essence, adhere better to their rehabilitation practices. However, they need to be familiar enough with the game systems and movements for them to attribute poor performance with their own lack of skill rather than an external device, which would otherwise deter them from continuing to play the game.

### Helplessness as a Barrier

Regarding RQ1, we found that a significant barrier to using the integrated wearable glove is the feeling of helplessness, particularly if it is in the context of technical difficulties. While the participants mostly agreed that the exergame navigation was easy to understand because of the simple nature of the menu layout and user-friendly, large, and visible buttons, the older adults were not familiar with the movement system of the wearable gloves. Therefore, while they had very clear intentions regarding navigating the menu, the disconnect between intention and execution made the process a lot more difficult than intended for the participants. This became even more apparent when participants were asked to describe if anything was confusing about the gameplay movements of Chinatown Race, to which many expressed the following general sentiment:

Not confusing, only unfamiliar with the controls [in reference to glove].

Participant 2; without a history of stroke

Therefore, it was evident to a certain degree that the overall experience of playing the game was hindered because the gameplay affordances provided by the glove apparatus were not intuitive to the participants. This, in turn, had a demotivating effect on the participants as their ultimately poor gameplay would then be attributed to an external factor such as the glove and the game system, making them feel somewhat helpless as they struggled to competently dodge all the obstacles and collect the harder-to-collect lantern items that required them to hyperextend their thumb. This is exemplified when looking at “necessity of support” (ie, question 4 of the SUS scale), where the participants were asked to rate how much technical support they would require to use the gloves. The patients with a history of stroke were more likely to want to get more help (mean 2.45, SD 1.51) as compared to those who had no history of stroke (mean 3.45, SD 1.37). This could be attributed to how older adults with a history of stroke may have experienced feelings of helplessness using novel rehabilitation technologies in the past and therefore might show an aversion to new technologies.

RQ2 deals with the differences between older adults without a history of stroke and those with a history of stroke regarding their perceptions about the glove integrated with exergame. Our findings indicated that the rehabilitation history influenced the level of critique from user groups when engaging with the integrated glove. The comfort level of the glove was a major theme; in the SUS questions, participants who did not have a history of stroke indicated that comfort level was very important (mean 3.78, SD 0.97) as compared to those with a history of stroke (mean 4.18, SD 0.60).

When asked about what could be improved to make the overall experience of playing Chinatown Race using the wearable gloves better, there was a noticeable difference regarding the category of improvement suggested by both groups. The comments by participants with a history of stroke tended to pertain toward making the controls more intuitive:

Improve the controls, like grabbing action for the lantern instead of thumb extension.

Participant 4; with a history of stroke

In contrast, comments by participants who did not have a history of stroke were more likely related to areas such as game design and esthetics (collectible items or general color contrast):

Barrier needs to be bigger in size and have more contrasting colours so that it can be seen from the distance.

Participant 19; without a history of stroke

This difference could suggest that older adults with a history of stroke were more focused on the movements associated with the gloves and what their actual bodies were doing in relation to the game than whether the game was appealing enough to be played effectively. A possible attribution for this difference could be how the older adults with stroke have an acute awareness that the exergame is a tool to be used in a long and tedious process that they have experienced previously, and therefore, they have deeper awareness of what is essentially important in the actual process of rehabilitation. In contrast, the older adults without a history of stroke could be looking at the exergame and the wearable gloves as just another gaming device, and therefore, their criticism would be directed toward the exergames as a game than as a rehabilitation tool.

Finally, for RQ2, our results showed that the valence of anticipation regarding using a new device varies based on their history with stroke. This is seen when looking at “intention to frequently use,” which refers to question 1 of the SUS scale used in the survey. It must be noted that the group without a history of stroke indicated more eagerness to use the glove with exergame (mean 4.38, SD 0.71) as compared to those with a history of stroke (mean 3.18, SD 1.47). It could be because participants who have had stroke before maintained a level of cynicism toward such novel methods for stroke rehabilitation as they have been through the rehabilitation process previously. Furthermore, it might be due to barriers related to perceptions of the ease of use. Participants without a history of stroke indicated that the glove was easy to use (mean 4, SD 1.50), and the score was slightly higher than those with a history of stroke (mean 3.64, SD 1.50). It might also be due to the visual esthetics, as there is a “box” attached to the glove that contains the wiring. A participant noted the following:

The box is big and is not needed...The extra electronic devices seem delicate and can break.

Participant 9; with a history of stroke

While the box itself is safely secured and presents no actual hindrance to the gameplay experience, the apparently excessive amount of gadgetry can scare older adults who are not familiar with the durability that most present-day technology have and, at worst, can incite a certain fear response within them to abstain from handling something they think can break any minute.

## Discussion

### Principal Findings

Several key findings were generated from this study. First, this study addresses the problem of data absenteeism and technology chauvinism by engaging older adults— those with and those without a history of stroke—to provide insights into the potential motivations and barriers regarding the use of integrated wearable glove with exergame solution for rehabilitation and exercise at the early stages of the development process. Most notably, while technologies such as wearable gloves and exergames play a pivotal role in the rehabilitation process, it is reductionistic to assume that the technology itself would be the panacea to addressing health issues. For instance, although older adults with and those without a history of stroke indicated that the glove and exergames were well integrated, we found that individuals who have a history of stroke were more likely to indicate that they would still require help in operating the wearable glove compared to those who have not experienced stroke, which was perceived to be complicated. This finding is supported by the “blind faith” aspect of technology chauvinism research: if technology is offered as a solution in isolation without paying attention to the larger social context of the participants, it would not be effective [13]. This is supported by our findings where older adults with a history of stroke indicated that they would need the support of a technical person to be able to use it effectively and that it could be unnecessarily complex. Thus, solutions that embrace the development and implementation of wearable gloves with exergames need to consider designing the technology such that it can be used in a community setting by tapping into the social and support networks of older adults as co-users to improve adherence and uptake. This is consistent with the study by Proulx et al [38], where they examined occupational therapists’ perceived usability and utility of a similar wearable glove for rehabilitation. In their study, while the researchers found that the occupational therapists rated the usability of such gloves ranging from “moderate to good” on the SUS, they shared that the gloves would be challenging for patients if they did not have the assistance of a therapist, owing to the physical and cognitive deficits of patients with stroke. The therapists also suggested that the development of wearable gloves would need to account for different contexts, such as using the gloves with therapists or with the assistance of a caregiver.

Second, it was noted that although older adults indicated that the gloves were relatively easy to use and that they intend to use them frequently, the comfort level while using the glove is an important factor for older adults with a history of stroke. This is corroborated by existing studies of technology acceptance and usability, which suggests that the practicality of use is a fundamental cornerstone in the acceptance and integration of technology into a daily routine. Our finding is consistent with that of the study conducted by Yurkewich et al [21], where they designed the HERO Grip Glove to help patients with stroke to perform activities of daily living and finger movement. While participants reported that they were relatively satisfied with the glove in terms of safety, security, and general ease of use, they had the lowest satisfaction regarding the ease of wearing the glove.

Third, we found that it is crucial to consider how gameplay could motivate or demotivate older adults from using wearable gloves. Existing studies of commercial device-based hand rehabilitation for patients with stroke have shown that game-based training using wearable gloves was generally positive in improving hand function and that they would be received favorably as they would be perceived as entertaining [41]. However, our study showed that the design of gameplay would need to aim for a fine balance in managing the difficulty level for a diverse group of patients and players, such that players would find the game challenging enough to sustain their interest, but it would not be very complex to demotivate them. This is important because our results showed that some older adults could be easily demotivated by some of the gameplay scenarios, especially when they feel that they cannot achieve the objectives (ie, collecting lanterns and points and avoiding barriers) or when their perceived expectations of their personal performance do not match with their scores. This results in some of them being demotivated, not enjoying the gameplay, and feeling helpless as they felt that there was nothing they could do to improve their scores.

Finally, it could be observed that individuals with a history of stroke show a stronger aversion to using rehabilitative technology and would thus require more assistance and technical support. This is an interesting as it suggests that those with previous experience with such novel rehabilitative technology may have a predisposed resignation that they would not be able to fully use these new technological methods in their recovery and would thus rather heavily rely on some form of instruction or expertise in conducting the rehabilitation exercises. Patients with a history of stroke have experienced rehabilitation in the past and thus could experience resistance to new rehabilitation technology due to a bias or preference to use what they are already familiar with [42].

In the case of patients with a history of stroke, they might prefer to use something that worked for them in the past because there is precedence of that working. Thus, getting used to a completely new device in stroke rehabilitation presents a level of psychological uncertainty or risk perception associated with the new method and would thus cause them to feel like they may not be able to adapt or manage it efficiently without proper assistance [43].

### Implications

The theoretical implication of the findings from this study has shown that the motivation levels toward new technology among a homogenous group of people can differ depending on their personal experiences related to the purpose of the proposed technology. For instance, in this study, it was demonstrated that even among older adult patients, the mere experience of a stroke altered their perception toward the wearable gloves by a significant degree compared to those who did not have a history of stroke. Therefore, it is crucial that future studies consider medical conditions or individuals’ experiences when designing health technologies.

The practical implication of the findings of this study illustrates how future exergames can be properly and suitably designed for an aging audience, for example, ensuring that visual elements can be differentiated from one another in a very clear manner and that the video game’s difficulty level is attuned not to be extremely difficult but moderately challenging to both prevent the demotivation of the players and encourage continued gameplay, which basically means that the patients continue the rehabilitation process through the exergame medium. In addition, digital health technologies must be designed to have clear affordances without visibly looking like it would be very difficult or complicated to use, as this has an effect on the users’ perception about the apparatus, which would inadvertently affect their motivation to buy and use the product.

### Limitations

Similar to all studies, there are several limitations in our research. The first limitation of this study would be the lack of a substantial sample size. The sample size of 19 is very small to draw concrete quantitative conclusions and comparison between the focus groups, and therefore, any findings and results that were gleaned from the study are educated guesses and conjecture. However, it should be noted that the opinions shared by the older adults during the study are ideas that people in the target group actually have, but they need to be carefully examined in future studies. Second, our participants only used the wearable gloves and games in 1 session with 2 games. We are cognizant that our participants who willingly participated in our research may be qualitatively different from those who did not join. Third, we are mindful that older adults who did not have a history of stroke might have other health conditions that the research team is not aware of that might influence their perceptions about the wearable glove and gameplay.

### Conclusions

In summary, there is tremendous potential in the use of digital health technologies such as wearable gloves and exergames to motivate older adults to exercise and, for patients recovering from stroke, to adhere to rehabilitation exercises. While we recognize the benefits of such digital health technologies, without representation from older adults in such studies, any technology development and implementation may face the problem of data absenteeism and technology chauvinism. Thus, to achieve a more equitable and inclusive use of digital health technologies, researchers need to consider both the individuals and the contexts in which the technologies are used.

## Appendix

Multimedia Appendix 1 Mean scores of the System Usability Scale items.

## Abbreviations

HERO: Hand Extension Robot Orthosis
RQ: research question
SUS: System Usability Scale
