# Supplementary figures and images for: Addressing Data Absenteeism and Technology Chauvinism in the Use of Gamified Wearable Gloves Among Older Adults: Moderated Usability Study
Source: JMIR Serious Games. 2024 Apr 24;12:e47600. doi: 10.2196/47600 (PMC11079763; doi:10.2196/47600)

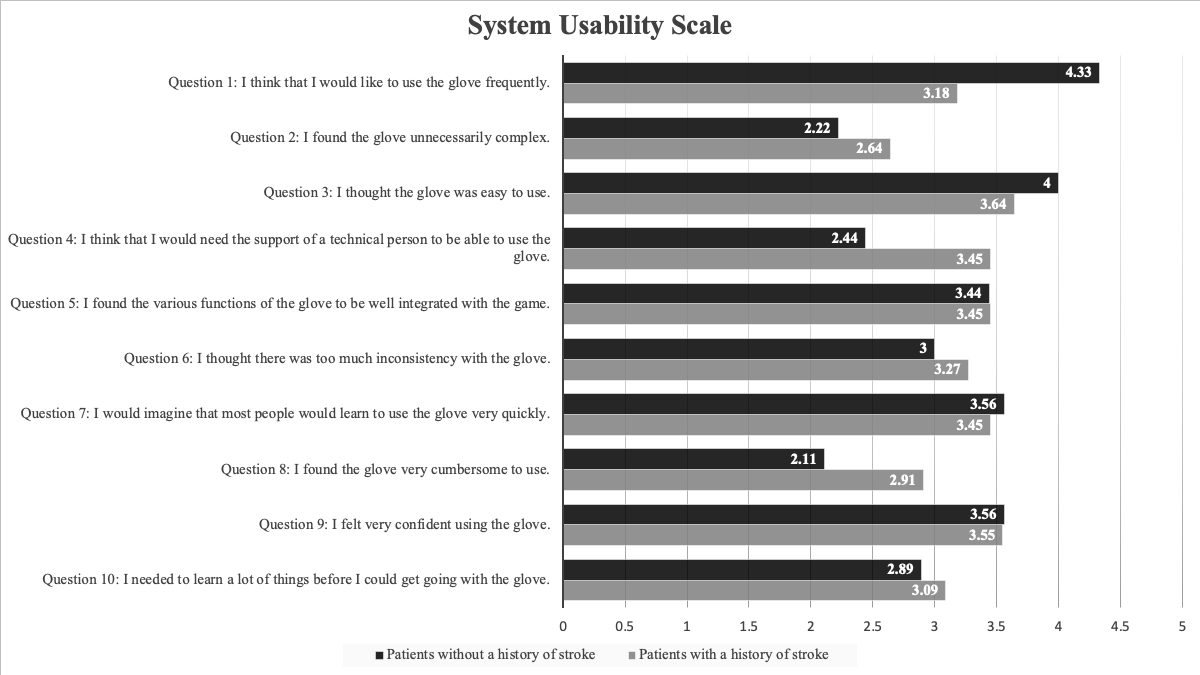

Supplement: Multimedia Appendix 1 [file games_v12i1e47600_app1.png]
